# Supplementary material for: Preoperative binaural beats reduce remimazolam dosage and enhance safety in anesthesia induction: A randomized controlled trial
Source: PLoS One. 2026 Mar 30;21(3):e0345960. doi: 10.1371/journal.pone.0345960 (PMC13035112; doi:10.1371/journal.pone.0345960)
Supplement: S1 File — (DOCX) [file pone.0345960.s001.docx]

**S1 File. Study protocol**

**Study title**

Preoperative binaural beats reduce remimazolam dosage and enhance safety in anesthesia induction: a randomized controlled trial

**Study design**

This study was a randomized, prospective, double-blind, single-center, two-arm clinical trial conducted at Yonsei University Gangnam Severance Hospital, Seoul, Republic of Korea.

**Ethical approval and trial registration**

The study was approved by the Institutional Review Board of Yonsei University Gangnam Severance Hospital (approval number: 2023-0759-001) on October 6, 2023.
The trial was registered at ClinicalTrials.gov (NCT06099977) on November 1, 2023.
Written informed consent was obtained from all participants prior to enrollment.

**Participants**

Eligible participants were adults aged 20–60 years with an American Society of Anesthesiologists (ASA) physical status of I or II, an ideal body weight between 50 and 80 kg, and scheduled for elective surgery under general anesthesia.

Exclusion criteria included hearing impairment; use of opioids or sedatives within one week prior to surgery; alcohol or drug dependence; hypersensitivity to remimazolam; arrhythmia; cardiovascular disease; heart failure; hypovolemia; or liver failure.

**Randomization and blinding**

Participants were randomly allocated in a 1:1 ratio to either the binaural beats group or the control group using a computer-generated randomization list. Group assignments were concealed in sealed opaque envelopes.

The application of headphones and auditory intervention was performed by a nurse who was not involved in patient care or outcome assessment. Anesthesiologists and outcome assessors were blinded to group allocation.

**Intervention**

Participants in the binaural beats group listened to real-time binaural beats with a 1-Hz frequency difference (431 Hz in the left ear and 432 Hz in the right ear) through headphones for 30 minutes in the anesthesia pretreatment room.

Participants in the control group wore identical headphones for 30 minutes but received no auditory stimulation.

**Anesthesia induction and management**

Standard monitoring included non-invasive blood pressure, electrocardiography, pulse oximetry, and Patient State Index (PSI) monitoring using the SedLine® brain function monitor (Masimo, Irvine, CA, USA).

After preoxygenation with 100% oxygen, remimazolam was administered by continuous intravenous infusion at a rate of 6 mg/kg/h until loss of consciousness (LoC). LoC was was defined as the absence of response to standardized verbal commands. During remimazolam infusion, verbal commands (“Please open your eyes”) were delivered every 5 seconds by the attending anesthesiologist using a predefined script. LoC was determined as the first time point at which the patient failed to respond to two consecutive commands. This protocol was applied consistently across all participants to ensure standardized assessment of LoC.

Following LoC, remifentanil infusion was initiated with a target effect-site concentration of 4 ng/mL using the Minto pharmacodynamic model, and rocuronium (0.8 mg/kg) was administered to facilitate tracheal intubation. Anesthesia was maintained with sevoflurane, remifentanil, and rocuronium.

**Outcome measures**

The primary outcome was the total dose of remimazolam required to achieve LoC.

Secondary outcomes included:

- Time to LoC
- Anxiety scores before and after headphone application
- Patient State Index (PSI) values at predefined time points
- Electroencephalography (EEG) spectral power changes
- Incidence of hypotension within 30 minutes after anesthesia induction

Hypotension was defined as a mean arterial pressure <65 mmHg or a decrease ≥20% from baseline.

**Data collection**

Physiological data, including blood pressure, heart rate, PSI, and EEG parameters, were continuously recorded using the SedLine® monitor and extracted using VitalRecorder software (version 1.13.9).

EEG spectral power was analyzed across standard frequency bands (alpha, beta, delta, gamma, and theta) before and after anesthesia induction.

**Statistical analysis**

Sample size calculation was based on an anticipated 20% reduction in remimazolam dose in the binaural beats group, with a power of 80% and a two-sided significance level of 0.05.

Statistical analyses were performed on an intention-to-treat basis using SPSS (version 25; IBM) and R software (version 3.6.1). Continuous variables were expressed as mean ± standard deviation, and categorical variables as counts and percentages.
