# Supplementary material for: Preoperative binaural beats reduce remimazolam dosage and enhance safety in anesthesia induction: A randomized controlled trial
Source: PLoS One. 2026 Mar 30;21(3):e0345960. doi: 10.1371/journal.pone.0345960 (PMC13035112; doi:10.1371/journal.pone.0345960)
Supplement: S2 File — (DOCX) [file pone.0345960.s002.docx]

**전향적 임상연구 계획서**

**1. 연구 제목, 단계, 계획서 식별번호 및 제·개정이력 등**

1) 연구 제목

연구 제목 (국문): 수술 전 시작한 양이성 음향이 전신마취 유도에 필요한 레미마졸람 용량에 미치는 영향: 무작위 대조 시험

연구 제목 (영문): Preoperative Binaural Sounds Reduce Remimazolam Dosage Required for Loss of Consciousness in General Anesthesia Induction: a Randomized Controlled Trial

2) 단계: 해당 없음

3) 계획서 식별번호: Protocol No. Binaural beats Remimazolam

4) 제·개정이력: version 1.1

5) 연구진 소속, 직위, 성명:

연구 책임자:

강남세브란스병원 마취통증의학과 임상부교수 김현창

연구 담당자:

강남세브란스병원 마취통증의학과 임상부교수 이지원

6) 연구 수행 장소 및 기간

• 연구 수행 장소: 강남세브란스병원 수술실, 회복실

• 연구 기간: IRB 통과 후 24개월

**2. 연구계획서 요약**

| 연구목적 | 수술 전 시작한 양이성 음향이 전신마취 유도를 위한 의식 소실에 필요한 레미마졸람 용량을 줄일 수 있는지 알아보고자 한다. |
| --- | --- |
| 연구설계 개요 | Prospective randomized controlled study |
| 목표 대상자 수, 산출근거 | 72명 본 연구에서 primary outcome로 설정한 마취 유도 시 필요한 레미마졸람 용량이 전향연구에서 평균 17.8 mg, 표준 편차 5.1 mg으로 조사되었다.  양이성 음향을 적용하여, 레미마졸람 용량이 3.56 mg (20% ) 감소하면 효과가 있는 것으로 가정하였을 때, 군당 33명이 필요하였다. 추적소실율 10%, 순응도 100%로 하였을 때, 각 36 명씩 72명의 연구대상자가 필요하다. |
| 선정. 제외기준 | 선정기준:  ① 전신마취를 요하는 만 20-60세의 성인 환자  ② 임상 시험에 참여하기 위한 동의서를 서면으로 제공할 수 있고, 본 임상 시험의 절차를 이해할 수 있어야 함  ③ 미국마취과학회 신체등급 분류 (ASA physical status classification) 1-2 등급  ④ 이상 체중 50kg에서 80kg 인 환자  남성 (kg): 50 + (0.91 × [height in centimeters − 152.4])  여성 (kg): 45.5 + (0.91 × [height in centimeters − 152.4])  제외기준  ① 난청이 있거나 보청기를 사용중인 환자  ② 1주 이내에 마약성 진통제나 진정을 위한 약물을 투여받은 환자  ③ 알코올 의존이나 약물 의존이 있는 환자  ④ 레미마졸람에 약물과민증이 있는 환자  ⑤ 부정맥, 심혈관 질환, 심장기능 저하, 순환혈류량 저하가 있는 환자  ⑥ 간부전이 있는 환자  ⑦ 이외 시험자 또는 시험 담당자의 소견에 따라 본 임상 시험에 적절하지 않은 것으로 판단되는 임상 시험 대상자 |
| 연구방법 | 본원과 전신마취 하 수술을 받는 만 20-60세 성인 환자들 중 본 연구의 참여에 동의한 환자 72 명을 무작위로 두 군으로 1:1 무작위 배정한다.  수술장 입실 전 수술장 입구에 환자가 도착하면 anxiety 점수를 visual analogue scale(VAS) 점수로 평가하고 양이성 음향 장치를 착용시킨다.  무작위로 배정된 배정표에 따라 시험군과 대조군으로 나누고 시험군에서는 양이성 음향을 30분간 적용시키고, 대조군에게는 무음상태를 30분 동안 적용한다. 수술방에 입실 후 심전도, 맥박산소포화도, 혈압계, sedline을 부착하여 활력징후, 마취 심도 감시를 시작한다. 마취유도 전 환자의 anxiety score를 visual analogue scale 로 평가한다. 마취유도를 Preoxygenation을 충분히 하고, 레미마졸람을 6 mg/kg/hr의 속도로 지속정주한다.  목소리(눈떠 보세요)에 반응이 없어질 때까지, 환자의 속눈썹 반사가 없어질 때까지, patient state index (PSI)가 50에 도달할 때까지 투여된 레미마졸람의 양, 시간을 평가한다. 신경근차단제를 투여하고 이후 마취과정은 양군 모두 동일하게 진행한다. Sedline 센서를 통해 뇌파의 raw data를 저장하고, 추가적으로 분석 평가한다. |
| 평가변수 | 일차평가변수  목소리에 반응이 없어질 때까지 투여된 레미마졸람 용량  이차평가변수  속눈썹 반사가 없어질 때까지 투여된 레미마졸람 용량, 시간, PSI 가 50이하로 떨어질 때까지 투여된 레미마졸람 용량, 목소리에 반응이 없어질 때까지 투여된 레미마졸람 체중 1kg 당 용량, 속눈썹 반사가 없어질 때까지 투여된 레미마졸람 체중 1kg 당 용량, 수술입실 전후 불안정도 평가, sedline 뇌파 |
| 자료분석 및 통계방법 | 자료는 정규분포를 따르는 연속형 자료에 대해서는 평균 ± 표준편차, 비정규분포를 따르는 자료에 대해서는 중앙값 (IQR) 등을 기술한다. 비연속형 자료에 대해서는 빈도 (%)를 기술한다. Primary outcome인 목소리에 반응이 없어질 때까지 투여된 레미마졸람 용량에 대해서 정규분포를 따르는 연속형 자료의 경우에서는 t-test를 사용하고, 비정규분포를 따르는 연속형 자료의 경우에서는 Mann-Whitney U test를 이용한다. 각 검정의 유의수준은 5%를 기준으로 한다 |

**3. 연구 배경 및 이론적 근거**

전신마취와 진정에 사용되고 있는 레미마졸람은 다른 마취제에 비해 저혈압의 빈도가 낮다.(1) 그러나 레미마졸람도 고용량일수록 또, 환자의 나이가 많을수록 저혈압을 일으킬 수 있다.(2) 가능한 적은 용량의 레미마졸람으로 전신 마취를 위한 의식 소실을 일으킬 수 있다면 보다 안전한 마취를 제공할 수 있을 것이다.

사람의 뇌파는 활성도와 정신상태에 따라 다른 주파수를 보이는데, 이러한 뇌파를 이용하여 마취 깊이를 감시할 수 있다. 이를 반대로 이용하는 것이 뇌파 동기화 방법이다. 이는 의료진이 지향하는 뇌파 주파수와 사람의 뇌파 주파수를 공명시켜 그 주파수 영역대의 정신상태를 유도하는 방법이다. 뇌파 동기화를 위해 사용되는 자극 중 양이성 음향이 있다. 양이성 음향은 양쪽 귀에 서로 다른 주파수의 소리를 들려줌으로써 간섭주파수로 뇌파를 공명시키는 뇌파 동기화 방법이다. 양이성 음향을 적용하여 이완기 혹은 휴식기에 나타나는 낮은 주파수의 음향을 전달했을 때, 대상자에게서 이완, 진정, 통증, 불안 등의 완화 효과가 있는 것으로 알려져 있다.(3, 4) 하지만, 레미마졸람을 이용한 전신마취 유도 과정에서 양이성 음향을 이용한 뇌파 동기화 방법을 알아보는 임상연구는 아직 없다.

이에 본 연구자는 수술 전 양이성 음향을 들려주는 것이 전신마취 유도에 필요한 레미마졸람 용량을 감소시키는지 알아보고자 한다.

**4. 연구 목적**

수술 전 시작한 양이성 음향이 전신마취 유도를 위한 의식 소실에 필요한 레미마졸람 용량을 줄일 수 있는지 알아보고자 한다.

**5. 위험/이익 분석**

잠재적 위험

본 연구에서 시행하는 중재는 헤드폰을 이용한 양이성 음향을 들려주는 것이기 때문에 발생하는 위험성은 미미할 것으로 생각된다. 이 외에 다른 수술, 마취는 연구에 참여하지 않는 환자와 동일하게 시행될 예정이다.

잠재적 이익

본 연구의 결과를 바탕으로 양이성 음향이 마취유도를 위한 레미마졸람의 용량을 줄인다면 향후 레미마졸람을 이용한 마취 유도를 받는 환자들에게 임상적으로 도움이 될 것으로 생각된다.

위험/이익 분석

환자는 전신 마취 유도가 전문의의 관찰 및 관리를 받게 되어 안전성이 증가되어 이익이 클 것으로 예상된다.

**6. 목표 대상자 수 및 산출 근거**

본 연구에서 primary outcome은 목소리에 반응이 없어질 때까지 투여된 레미마졸람 용량이다. 전향연구에서 목소리에 반응이 없어질 때까지 투여된 레미마졸람 용량은 평균 17.8 mg, 표준 편차 5.1 mg으로 조사되었다.

양이성 음향을 적용하여, 레미마졸람 용량이 20% (3.56 mg) 감소하면 효과가 있는 것으로 가정하였을 때, 군당 33명이 필요하였다. 추적소실율 10%, 순응도 100%로 하였을 때, 군당 36명, 총 72명의 연구대상자가 필요하다.

**7. 대상자의 선정/제외 기준 (중도탈락 기준 등)**

1) 선정 기준

① 전신마취를 요하는 만 20-60세의 성인 환자

② 임상 시험에 참여하기 위한 동의서를 서면으로 제공할 수 있고, 본 임상 시험의 절차를 이해할 수 있어야 함

③ 미국마취과학회 신체등급 분류 (ASA physical status classification) 1-2 등급

④ 이상 체중 50kg에서 80kg 인 환자

남성 (kg): 50 + (0.91 × [height in centimeters − 152.4])

여성 (kg): 45.5 + (0.91 × [height in centimeters − 152.4])

2) 제외 기준

① 난청이 있거나 보청기를 사용중인 환자

② 1주 이내에 마약성 진통제나 진정을 위한 약물을 투여받은 환자

③ 알코올 의존이나 약물 의존이 있는 환자

④ 레미마졸람에 약물과민증이 있는 환자

⑤ 부정맥, 심혈관 질환, 심장기능 저하, 순환혈류량 저하가 있는 환자

⑥ 간부전이 있는 환자

⑦ 이외 시험자 또는 시험 담당자의 소견에 따라 본 임상 시험에 적절하지 않은 것으로 판단되는 임상 시험 대상자

**8. 스크리닝 검사 항목 및 방법**

1) 환자의 과거력과 내, 외과적 기왕력

2) 수술 전 흉부 방사선 검사, 말초혈액검사, 전해질검사, 혈당검사, 소변검사 등

- 스크리닝 검사 항목은 수술 전 일반적으로 시행하는 것으로, 이 연구를 위해 추가로 시행하는 검사는 없다.

**9. 연구 설계 및 방법**

**1) 구체적인 연구방법**

**Preoperative period**

수술실 입실 전, 수술장 입구에 환자가 도착하면 anxiety score를 visual analogue scale (VAS) 로 (0-10) 평가하고(5) 헤드폰 (현재 구비가 되어 있는 개인 물품)을 착용시킨다. 헤드폰은 양이성음향을 적용시킬 수 있는 모델이다. 이 때 실험과 무관한 제3자 (연구에 참여하지 않는 마취통증의학과 간호사)가 무작위 배정표를 보고 시험군에게는 양이성 음향을, 대조군에게는 무음상태(무음상태의 음향파일) 를 30분 이상 적용한다. 음향발생기기는 애플 아이폰13프로 모델 (현재 구비가 되어 있는 개인 물품)이며, 침대의 수액걸이에 걸어서 환자와 같이 이송된다. 음향은 양이성음향의 경우 양쪽 귀에서 1~4 Hz를 달리하여 맥놀이에 의해 간섭파를 만들 수 있게 하며, 배경음악은 없으며, 음량은 음향발생기기에서 기본으로 송출되는 음량보다 1단계 낮은 레벨로 하여 시작한다. (60dB) 헤드폰은 일회용 커버를 사용하며, 사용 후 헤드폰을 소독 솜으로 소독한다. (일회용 커버 및 소독 솜은 연구자가 부담합니다.)

**Intraoperative period**

수술장 입실 후 심전도, 맥박산소포화도, 혈압계, sedline을 부착하여 활력징후, 마취심도(PSI, patient state index) 감시를 시작한다. 정맥관의 가장 근위부에서 3 way 혹은 약을 주입할 수 있는 커넥터를 연결하여 준비한다. 음량을 0으로 바꾼 후 헤드폰을 벗기고, 마취 전 Anxiety score를 VAS 로 평가한다.

산소 8L/min으로 Preoxygenation을 충분히 하고, 레미마졸람을 6 mg/kg/h 로 지속정주하기 시작한다. 레미마졸람 투여는 의식 소실을 확인할 때까지 투여한다.

목소리(눈떠 보세요)에 반응이 없어질 때까지, 환자의 속눈썹 반사가 없어질 때까지, PSI가 50에 도달할 때까지 투여된 레미마졸람의 양, 시간을 평가한다. 3가지 평가(목소리에 반응이 없어짐, 속눈썹 반사 소실, PSI ≤ 50) 중 3가지 모두 양성일 때까지 레미마졸람을 투여한다. 각 평가까지 투여된 레미마졸람을 기록한다. 이 때 Desflurane흡입, Remifentanil 목표처 농도 4ng/mL로 주입을 시작하고 신경근차단제를 투여한다. 이후에는 레미마졸람은 투여하지 않는다. 마취유도 중 PSI가 25-50 사이에 있도록 흡입마취제의 농도를 조절하고, 이후 마취과정은 양군 모두 동일하게 진행한다.

지속적으로 혈압과 맥박, PSI를 감시하고, 기관 삽관 직후의 흡입마취제의 MAC 값과, 활력징후, Psi를 기록한다.

Sedline 센서를 통해 뇌파의 raw data를 저장하고, 추가적으로 분석 평가한다. 1차 유효성 평가변수는 목소리에 반응이 없어질 때까지 투여된 레미마졸람의 용량이다.

**10. 연구 절차 및 평가**


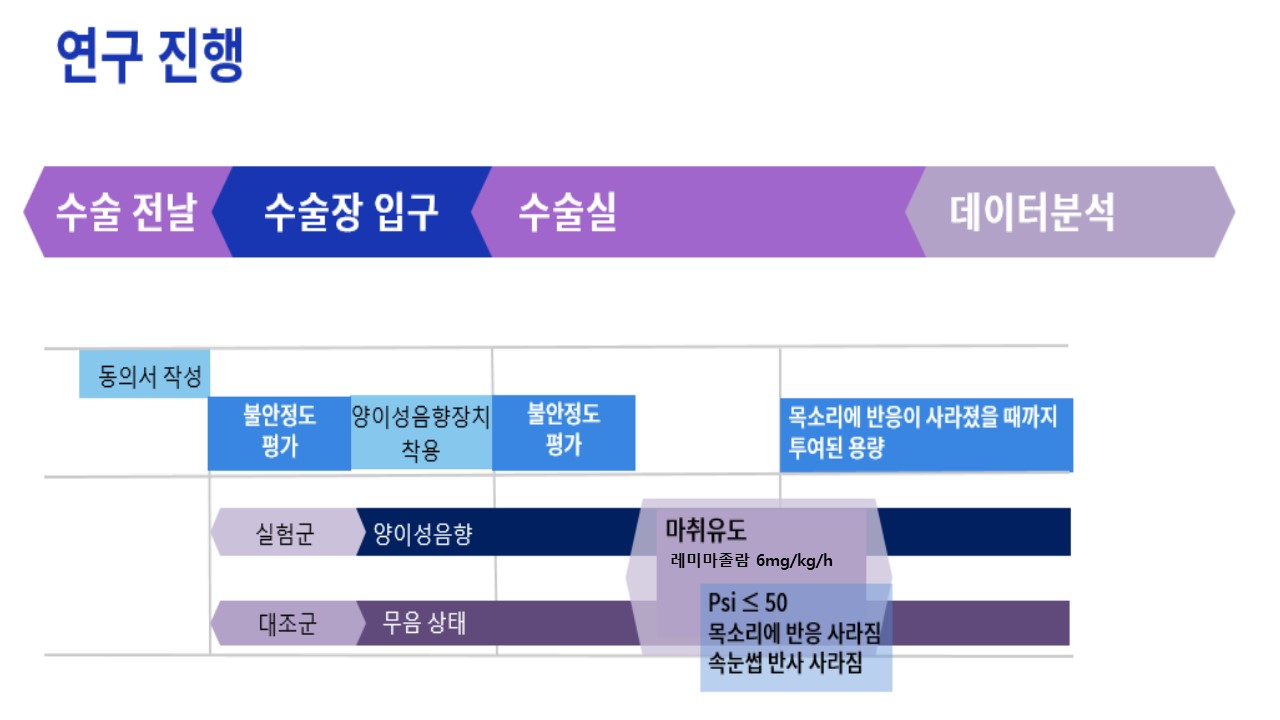


**11. 종료 및 조기 중단 기준**

1) 연구 종료: 연구 계획대로 절차가 모두 완료된 경우

2) 연구의 조기 중단

- 연구 기간 중 부작용 혹은 중대한 위해 요소 등으로 연구를 유지할 수 없는 경우

- 기타 연구진의 판단에 의해 연구 중지가 필요한 경우

**12. 이상반응을 포함한 안전성의 평가기준, 평가방법 및 보고 방법**

본 시험에 참여한 모든 환자를 대상으로 안전성 평가를 실시한다. 연구를 진행하는 동안 환자의 심전도, 혈압, 맥박산소포화도, 호기 말 이산화탄소 분압 등을 지속적으로 감시한다. 연구시행 중에 이상반응이 발생했을 경우, 연구에 사용된 시험 방법과의 인과 관계 유무와 모든 이상 반응을 기록하고 추후 중증도, 중대성, 기간, 그리고 시험 방법과의 인과관계를 평가한다. 이상반응에 대한 처치 및 결과 역시 기록한다. 이상반응의 빈도, 발현율, 각각의 목록, 심각한 정도 및 시험 방법과의 인과관계 등을 제시하며, 필요한 경우 그래프 형태로 보고한다.

① 중증도

이상반응은 아래의 정의에 따라 경증, 중등증, 중증으로 구분된다. 경증은 일반적이고 일시적이고 일상적인 활동을 방해하지 않습니다. 중등증은 약간의 불편함을 초래하거나 일상적인 활동을 방해한다. 중증은 일상적인 활동을 수행할 수 없다.

② 인과 관계

인과 관계는 ‘관련 없을 것으로 생각됨’, ‘관련 있을 가능성 있음’,’가능성 많음’,’명백히 관련 있음’, 또는 ‘관련성을 확인하기 어려움’으로 구분된다.

③ 이상 반응의 분류

활력징후 불안정의 합병증이 발생할 수 있다. 이는 기존의 통상적인 전신마취 유도시에 발생할 수 있는 합병증이며, 본 연구로 인하여 추가적인 위험이 생길 여지는 적다.

**13. 자료분석 및 통계학적 고려사항**

모든 분석은 의도 대로 치료(intention-to-treat, ITT) 원칙에 따라 수행되었다. 연속형 변수의 경우, Shapiro-Wilk 검정을 통해 정규성을 확인하였다. 정규 분포를 따르는 경우 평균 ± 표준편차로 제시하고, 정규성을 따르지 않는 경우에는 Mann-Whitney U 검정을 사용하였다. 시간(마취 유도 전 vs. 후)과 그룹(양이성 음향 그룹 vs. 대조군)의 효과를 평가하기 위해 이원분산분석(two-way ANOVA)을 수행하였으며, 사후 분석(post-hoc)으로 Student’s t-검정 또는 Mann-Whitney U 검정을 적용하였다. 범주형 변수는 카이제곱 검정(chi-squared test) 또는 Fisher의 정확 검정(Fisher’s exact test)을 사용하여 비교하였다.

뇌파(EEG) 데이터는 SedLine® 뇌 기능 모니터와 VitalRecorder 소프트웨어(버전 1.13.9)를 사용하여 수집 및 분석되었다. 상대 뇌파 파워(relative EEG power)는 알파(8–12 Hz), 베타(12–30 Hz), 델타(0.5–4 Hz), 감마(30–100 Hz), 세타(4–8 Hz) 주파수 대역별로 총 뇌파 파워에 대한 백분율로 계산되었으며, 10초 간격으로 평균화되었다. 마취 유도 전 10분과 레미마졸람 주입 시작부터 의식 소실(LoC)까지의 데이터를 수집하여, 각 10초 간격의 총 파워를 기준으로 정규화(normalization)하였다. 상대 뇌파 파워의 변화는 마취 유도 후 값에서 마취 유도 전 값을 뺀 값으로 계산하였으며, 양수 값은 유도 후 상대 파워 증가를, 음수 값은 감소를 나타낸다. 그룹 간 차이(양이성 음향 그룹 값 - 대조군 값)는 95% 신뢰구간(confidence interval)과 p-값으로 보고되었다.

통계적 유의성은 p<0.05로 설정하였다. 모든 통계 분석은 SPSS(버전 25; IBM, Armonk, NY, USA)와 R 소프트웨어(버전 3.6.1; R Foundation for Statistical Computing, Vienna, Austria)를 사용하여 수행되었다.

**14. 개인정보보호 및 연구자료의 기밀 유지를 위한 방안**

연구 자료는 본 연구를 위한 목적으로만 사용되며 연구책임자, 연구담당자 외에 자료의 열람과 분석을 제한한다. 임상 연구 대상자의 개인식별 정보는 코드화 하여 screening sheet와 분리하여 기록하며, 자료는 잠금 장치가 있는 장과 접근이 제한된 컴퓨터에 보관하도록 한다.

그리고, 대상자는 본인의 개인 정보 수집 및 이용, 제공에 대한 수락 여부를 자유롭게 결정할 수 있다. 연구 관련 기록은 생명윤리법 시행규칙 제15조에 따라 연구가 종료된 시점부터 3년간 보관할 것이며, 보관 기간이 지나게 되면 개인정보보호법 시행령 제16조에 따라 파기할 예정이다.

대상자가 중도탈락 하는 경우, 탈락 이전까지의 수집된 정보는 연구에 사용될 수 있지만 더 이상 새로운 연구 정보를 수집하지는 않는다. 또한, 이 자료도 연구가 종료된 시점부터 3 년간 보관할 것이며 보관 기간이 지나면 파기할 계획이다.

**15. 인체 유래물, 유전정보 등 수집 시 관리·보관·폐기 방안**

해당 없음.

**16. 대상자 모집 방법 및 동의 절차**

선정 기준에 부합되는 연구대상자들을 대상으로 설명을 한 후, 수술 전날 혹은 1시간 이상의 충분히 생각할 시간 간격을 두고, 서면 동의를 받는다.

**17. 취약한 대상자 모집 시, 보호 방안**

해당 없음.

**18. 품질관리 및 신뢰성 보증 등 계획(자료 안전 모니터링 계획)**

1) 자료 및 안정성 모니터링 책임자: 이지원

2) 자료 및 안정성 정보 수집 및 검토 주기: 12개월 마다

3) 연구의 지속, 변경, 중단 결정을 위한 주요 유효성 평가 변수 및 검토 절차

- 책임연구자가 자료 검토 주기에 따라 동의서 확인, 대상자 안전성 검토, 근거 문서와 증례기록서 대조를 통한 자료 안전 모니터링을 실시한다. 본 연구와 관련하여 중대한 부작용과 같은 사항이 발생하는 경우 상황에 따라 연구를 변경하거나 중단을 논하고 회의 결과를 연구윤리위원회에 보고하여 후속 조치를 취한다.

4) 이상반응, 예상하지 못한 문제, 계획서 미준수 등에 대한 문제에 대해서 연구 담당자는 연구책임자에게 이를 보고하고, 연구책임자는 IRB에 보고한다.

**19. 연구실행 계획표 (일정표)**

1) 연구 계획 및 IRB 승인: IRB제출 이후 2개월 예상

2) 대상자 모집 및 연구 수행: IRB 승인 후 19개월 예정

3) 자료분석 및 결과보고: 1개월 예정

4) 논문 작성: 2개월 예정

**20. 참고문헌**

1. Wesolowski AM, Zaccagnino MP, Malapero RJ, Kaye AD, Urman RD. Remimazolam: Pharmacologic Considerations and Clinical Role in Anesthesiology. Pharmacotherapy. 2016;36(9):1021-7.

2. Chae D, Kim HC, Song Y, Choi YS, Han DW. Pharmacodynamic analysis of intravenous bolus remimazolam for loss of consciousness in patients undergoing general anaesthesia: a randomised, prospective, double-blind study. Br J Anaesth. 2022;129(1):49-57.

3. Huang R, Wang J, Wu D, Long H, Yang X, Liu H, et al. The effects of customised brainwave music on orofacial pain induced by orthodontic tooth movement. Oral Dis. 2016;22(8):766-74.

4. Padmanabhan R, Hildreth AJ, Laws D. A prospective, randomised, controlled study examining binaural beat audio and pre-operative anxiety in patients undergoing general anaesthesia for day case surgery. Anaesthesia. 2005;60(9):874-7.

5. Facco E, Stellini E, Bacci C, Manani G, Pavan C, Cavallin F, et al. Validation of visual analogue scale for anxiety (VAS-A) in preanesthesia evaluation. Minerva Anestesiol. 2013;79(12):1389-95.
