# Supplementary figures and images for: Preoperative binaural beats reduce remimazolam dosage and enhance safety in anesthesia induction: A randomized controlled trial
Source: PLoS One. 2026 Mar 30;21(3):e0345960. doi: 10.1371/journal.pone.0345960 (PMC13035112; doi:10.1371/journal.pone.0345960)

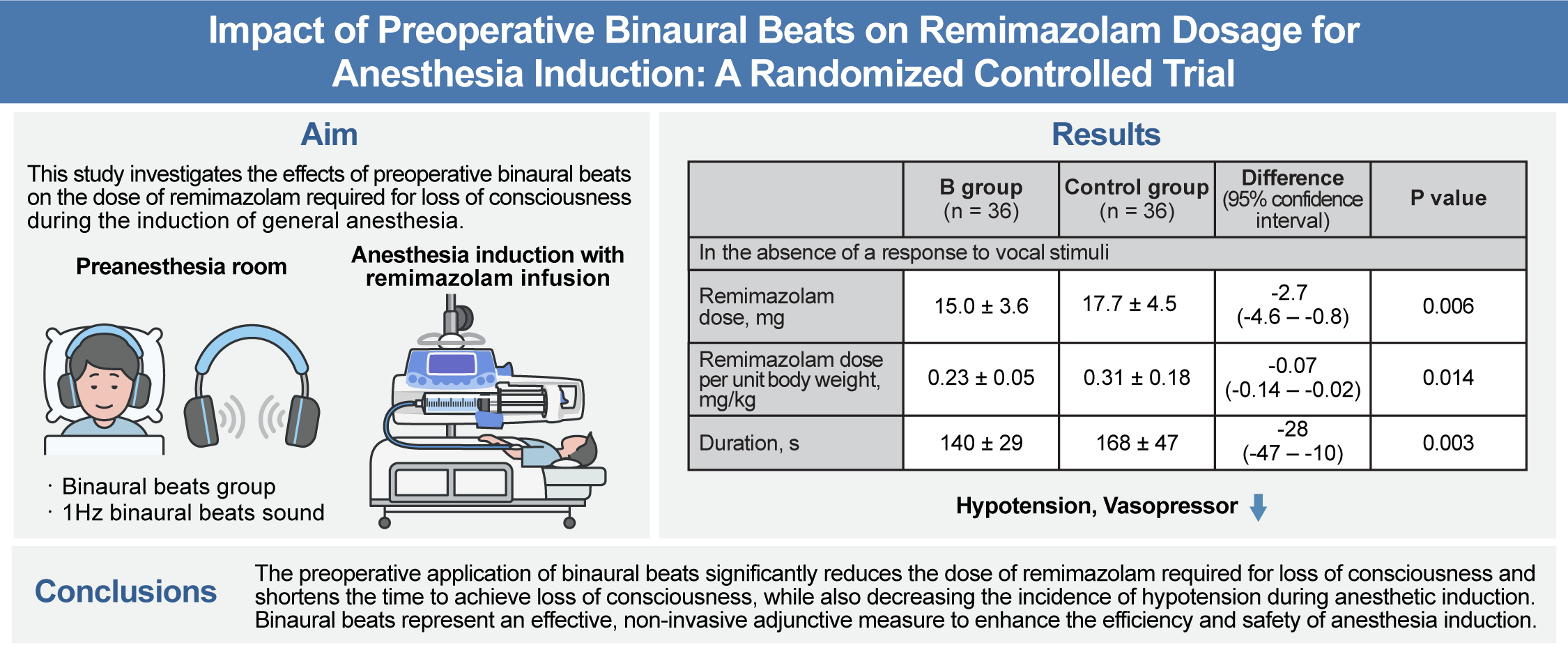

Supplement: S4 File — (TIFF) [file pone.0345960.s005.tif]
